# Mr. Semple's *Case of Abscess in the Liver*

**Published:** 1814-01

**Authors:** Robert Semple

**Affiliations:** Member of the Royal College of Surgeons in London, and Surgeon to the Parish of Islington. 35, High-street, Islington


*To the Editor of the Medical and Physical Journal.*

SIR,

THE following case occurred to a patient under my care in the work-house of this parish, the particulars of which I beg leave to relate.

Mrs. Thomas, aged 47 years, applied to me, a few days after after I entered on my office as surgeon to the work-house at Michaelmas last, for relief for a swelling of the abdomen, accompanied with obstinate constipation of the bowels. I prescribed some opening physic, which afforded relief, but the swelling remained the same. Upon inquiring into the nature of this complaint, I could gather no other information than that the woman was reported, and firmly believed to be, pregnant, which she obstinately denied. Her catamenia had ceased fifteen months ago, and she had borne several children. It appeared to me a case of interest, but I could procure no particular account of the progress of the swelling, only that it had appeared a twelvemonth ago, and had gradually increased to this period. On examination, I found the abdomen very much enlarged in size, with an evident fluctuation; and the swelling was by no means circumscribed, but general and diffused. She complained of no particular pain when the belly was pressed, and her health was tolerably good. She made water pretty freely, independently of which, I thought her disease was ascites, in which I was confirmed by the opinion of Mr. William Hole, a respectable surgeon in this town. We examined the patient together, and it was agreed that I should perform the operation of paracentesis of the abdomen. She complained of a little pain in the right shoulder, but none in the region of the liver. The operation being determined upon, I tapped her this morning (Nov. 1st) in the linea alba, with a moderate-sized trocar, and instead of water, I drew off, to my surprise, more than three quarts of very thick good-conditioned pus. I considered the disease now in a new light, and did not hesitate to pronounce it an abscess in the liver which had burst into the cavity of the abdomen. The matter was not at all fœtid. On pressing the region of the liver, after the trocar was withdrawn, she complained of pain. She had a stool after the operation, and made water freely. I closed the wound up, and applied a moderately tight bandage over the abdomen, and gave her forty minims Tinct. Opii and ℥iſs. Aq. Menth. Pip. in a draught. In four hours after the operation I saw her. She appeared tranquil, but had considerable pain in the region of the liver, extending to the right shoulder. Her pulse was firm and regular. I ordered her a little wine, and the draught repeated with only twenty minims of the Tinct. Opii.

Nov. 2d.—Passed a restless night; pulse about 90; thirsty; tongue foul; pain in the liver and right shoulder.

Hab. Pil. Hydrarg. gr. vi. horâ somni.

Nov. 3d.—Feels rather easier; bowels costive.

 ℞. Sulph.

℞. Sulph. Magnes. ℥i.

Aq. Menth. Pip.

—— puræ ā. ℥iv. M. Cap. Coch. iij. larg. omni horâ donec alvus respondeat.

Nov. 4th.—Had one black hard fœtid stool, which gave relief. Belly rather swelled; pain in the region of the liver; pulse firm. Her constant complaint being constipation, I ordered her the following, lest the circumstance above-mentioned should gain ground upon me.

℞. Pulv. Scammon. gr. v.

—— Jalap. ℈i.

Sub. Mur. Hydr. gr. x. M. ft. pulv. horâ 2da p. m. sumend.

Pil. Hydrarg. gr. vi. h. s.

Nov. 5th.—Had about a dozen black offensive copious motions, with much relief; T. pretty clean; makes water freely; pulse 100, quick, but regular; belly much softer, and feels easier; thirsty; slight pyrexia.

℞. Acid. Sulph. Dilut. ʒij.

Tinct. Digital. ʒi.

Aq. Menth. Pip. ℔i. M. Cap. coch. ij. larg. quater in die.

Nov. 6th.—Had two large offensive motions, partly watery, and mixed with scybalæ; abdomen rather softer, but still tumid; pulse 100, rather feeble; tongue furred; passed a restless night.

℞. Sub. Muriat. Hydrarg. ℈i.

Opii Pulv. gr. v.

Conserv. Ros. q. s. ut ft. massa in pilul. v. dividend. quarum capt. 1m. omni 4ta. horâ incip. statim.

Nov. 7th.—Slept well during the night, and without pain. Took all the pills, but had no motion from them. Tongue pretty clean; pulse 100; belly feels softer. In one of her motions of yesterday, we found several small gall stones. Upon the whole she seems amazingly better.

℞. Sulph. Sodæ. ℥ifs.

Infus. Sennæ.

Aq. Menth. Pip. ā. ℥iij. M. cap. dimid. statim alteramque partem post horas tres.

8th.—Had eighteen copious stools, mixed with scybalæ, and substances resembling clots of pus, and rather greenish. Pulse pretty good; tongue clean; belly much reduced in size, and softer.

℞. Pulv. Opii. gr. iij.
Sub. Muriat. Hydr. gr. x.
Pulv. Digital. Purp. gr. viij.

Conserv.

Conserv. Rosæ, q. s. ut ft. massa in pil. iv. dividend. quarum. cap. 1m. omni 4ta. horâ.

Nov. 9th.—Passed a good night, and free from pain. Had one copious loose stool before she took the pills, but none after. It was chiefly greenish clots, and a few scybalæ.

℞. Sulph. Sod. ℥i.

Aq. Menth. Pip. ℥vi. Solve. Capt. statim.

Nov. 10th.—Had eleven copious watery stools, in quantity more than half a gallon, mixed with a few scybalæ. Pulse 90, firm and regular; tongue clean; appetite good; belly much softer, and more natural.

Hab. Pil. Hydrarg. gr. vi. h. s.

Nov. 12th.—Feels pain in the bowels; one large motion since last report.

Repet. Mist. Purgans.

Nov. 13th.—Feels much better; four loose motions, and pretty natural.

Pil. Hydrarg. gr. vi. h. s.

Nov. 14th.—Had two copious loose motions; passed a very good night; pulse 88, regular; removed the plaisters, and found the wound completely healed, and free from inflammation; belly feels soft and flaccid, and free from pain; slight ptyalism; tongue clean.

Repet. Mistur. Purgans. u. a.

Nov. 15th.—Had four large loose stools of a natural color; slept well during the night; pulse 84.

Quiescat hodie.

Nov. 24.—Since last report my patient has progressively got better. She sits up through the day, and feels no pain or uneasiness. Her health is pretty good. She eats and sleeps well. I give her gr. iii of the Pil. Hydrarg. every third or fourth night, and shall gradually lay aside the use of medicine altogether.

If you think this case worthy a place in your Journal, you are at liberty to insert it.

I have the honor to be, Sir,

Your obedient Servant,

ROBERT SEMPLE,

*Member of the Royal College of Surgeons in London, and Surgeon to the Parish of Islington.*

*35, High-street, Islington,*

*Nov. 24,* 1813.

